# A Discussion of Positive Behavior Support and Applied Behavior Analysis in the Context of Autism Spectrum Disorder in the UK and Ireland

**DOI:** 10.1007/s40617-023-00905-x

**Published:** 2024-01-25

**Authors:** David Stalford, Scott Graham, Michael Keenan

**Affiliations:** 1 Ulster University-Coleraine Campus, Londonderry, Northern Ireland; 2 Ulster University, Coleraine, Londonderry, Northern Ireland

**Keywords:** Applied behavior analysis, Positive behavior support, Autism, Science, Aversive procedures

## Abstract

This article addresses the relationship between applied behavior analysis (ABA) and the emergence of positive behavior support (PBS) in context of autism spectrum disorder (ASD) in the UK and Ireland. Two overarching issues that are salient in this discussion are professional training and certification. To date, there has been a lack of standardized training or statutory requirements to practice PBS despite proponents insisting that its practice should be grounded in behavior analytic principles. Furthermore, there is an undercurrent of anti-ABA bias fueled by misinterpretation and unsubstantiated anecdotal claims used to promote an alternative “value based” approach to managing behavior.

Previous literature has discussed the differences and similarities between applied behavior analysis (ABA) and positive behavior support (PBS) and the associated implications (e.g., Brown et al., 2008; Johnston et al., 2006). The concerns discussed within the current article are by no means unique or new to our field, however, the concerns remain. It is alarming that within the United Kingdom (UK) and Ireland, it can be argued that the distinction between ABA and PBS is broadening, creating a “hard border” between the two camps. The purpose of the current article is not to regurgitate previous debates and discussions but rather to outline the current relationship between ABA and PBS within the UK and Ireland. In doing so, we will examine the outcome of these concerns insofar as they affect behavior services.

There are 700,000 autistic people in the UK which equates to 1 in 100 (Buescher et al., 2014). Specific to Northern Ireland (NI, 2021), governmental statistics consistently reveal that the number of individuals diagnosed with autism spectrum disorder (ASD) has substantially increased yearly over the last 2 decades. Northern Ireland Health and Social Care Service is divided into five public sector corporations or Trusts. Each Trust represents a particular geographical location within the region and are regarded as part of the UK’s National Health Service (NHS).

An examination of figures on all five Health Trusts revealed that 2,345 children under 18 years of age were diagnosed with ASD in 2018, compared with 1,047 in 2013 (NI, 2021). The latest update in NI from January to March 2022 reveals an overall figure of 2,563 children diagnosed with ASD. Thus, the upward trend is continuing. Furthermore, in terms of referrals, data from September 2002 to 2023, show that the number of children referred for autism services, across all five Trusts, has increased by 36% (Heidi & Kinghan 2023). This upward trend is reflected in prevalence rates in other countries (Anorson et al., 2021; Hai et al., 2023; Talantseva et al., 2023; Zeidan et al., 2022). Increasing prevalence of ASD inevitably leads to the need for increased provision of health and educational services (Bürki et al., 2021) with an associated financial burden on these service providers (Buescher et al., 2014; Grosse et al., 2021; Howlin & Moss, 2012). The estimated financial cost to the UK economy in 2011 was £32 billion (Buescher et al., 2014). These figures underscore the importance of government and charitable agencies making informed decisions on where to concentrate resources, such as staff training and professional development (Fennell & Dillenburger, 2018; Saigh & Bagadood, 2022).

Although ABA is not exclusively an intervention for ASD (Heward et al., 2022), its underlining methodology has provided numerous empirically validated interventions to assess and assist individuals with ASD (e.g., Boyle et al., 2019; Chiesa, 2005; Dillenburger & Keenan, 2009; Ghaemmaghami et al., 2021; Lovaas, 1987; Matson et al., 1996) and it has been recommended as providing the basis for addressing difficulties associated with ASD and intellectual disability (ID; see Larsson, 2021; Leaf & McEachin, 2016; Leaf et al., 2022). However, a public information site from a leading UK-based charity, the National Autistic Society (NAS, 2022), recently painted a different picture of ABA. The charity posted information about PBS and ABA on its web page. When referring to ABA, they implied that cruel punishment methods were currently being used in the UK by ABA practitioners; there was no evidence to substantiate the claim. Moreover, emotive terms such as “barbaric, cruel, traumatizing” (NAS, 2022, p. 1) were paired with a description of ABA, implying that such practices significantly contribute to people's opposition to ABA. The NAS stated they "do not support any intervention that follows one-size-fits-all approaches” and that they “believe that some ABA interventions used today are not sufficiently person-centered and are too intensive" (NAS, 2022, p. 1). Although the post concedes that "ABA is now one of the most researched of all autism approaches" (NAS, 2022, p. 1), there is a caveat that stated that there are significant limitations and gaps in the research, particularly about its long-term effects. With no clarification of where these gaps are, the statement implied that one possible long-term effect is the traumatization of those exposed to ABA interventions. It is paradoxical that although the NAS stated that support "should always be based on best available evidence" (NAS, 2022, p. 1), there is no mention of the lack of empirically validated research regarding the long-term effects of PBS. It is significant that the British Institute of Learning Disabilities (BILD), an organization that purports to provide information, publications, guidance, and training services to improve the quality of life for people with a learning disability, appeared to agree with the misinformation published by the NAS when they posted the following statement on its web page:

We know that some people are concerned that PBS is similar to Applied Behavior Analysis (ABA). At BILD, we are clear that PBS and ABA are not the same thing. The NAS has published a helpful statement highlighting differences between ABA and PBS. (BILD, 2022, p. 2)

Being clear that PBS is not the same thing as ABA is different from clarifying any similarities or differences. It is the contention of the current authors that this BILD statement gives the impression that they sanction this view of ABA and, by default, recommend PBS as the preferred approach for individuals diagnosed with ASD or ID. To the best of our knowledge, no organization has challenged the above statements made by NAS and BILD.

It is the current authors’ assessment that a primary problem is how the NAS and BILD characterize ABA as “an approach.” This sleight of hand stems from a category mistake that results in an applied science being placed on a par with specific interventions or branded packages of intervention (Chiesa, 2005; Cooper et al., 2020; Keenan & Dillenburger, 2018). The result is that ABA is wrongly categorized as a therapeutic intervention for ASD instead of being viewed as a practical application of a scientific discipline (Dillenburger, 2011). In addition to this issue, the NAS, although defining ABA as a broad approach that has changed over time, briefly described the origins of ABA by focusing on a narrow range of interventions without historical or theoretical context. Unfortunately, this uninformed narrative is not uncommon among opponents of ABA (e.g., Anderson, 2023; Milton, 2018). The possible implication of such misinformation/misrepresentation of ABA alongside the unabashed promotion of PBS to policymakers, or the general public, is of great concern to behavior analysts working and advocating for effective evidence-based intervention services to assist individuals with ASD (Keenan & Dillenburger, 2018). The rest of this article examines PBS and ABA both in a historical and present context with reference to peer-reviewed publications, research, and observations from the first two authors who work within the PBS framework/model. As stated, the concerns highlighted within the current article are not new to our field. However, rather than being resolved, the attempt to label ABA as the "bad boy of ASD interventions" appears to be gathering pace in UK and Ireland. For this reason, we feel it is important to rejuvenate this discussion.

## PBS Values and Emphasis

The increased prevalence of ASD seems to be paralleled by the ever-increasing number of behavior management strategies, and interventions, many of which claim to be evidence-based (Jacobson et al., 2005). Such a prevalence of often diverse procedures makes any analysis of the parameters and components contributing to efficacy a challenging process (Makrygianni et al., 2018). In the past few decades, PBS (more commonly referred to as positive behavior intervention support [PBIS] in the United States) has pervaded the areas of education, institutional, and community care provisions for individuals with a diagnosis of ASD and those with ID (Guercio & Murray, 2014; Kern & Lane, 2015; Storey & Post, 2019).

PBS can be defined as a method to increase an individual's repertoire utilizing methods of system changes to improve an individual's living environment; it is focused on quality of life in addition to providing solutions to challenging behavior (Horner et al., 1990; Koegel et al., 1996). The general perception of an overreliance on aversive behavioral change procedures to deal with challenging behavior attributed to early behavior modification or ABA interventions (LaVigna & Donnellan, 1986; Meyer & Evans, 1989; Will, 1999) is often linked to the inception of PBS. Thus, PBS emergence can be seen in the context of the move towards using less aversive forms of behavioral management of individuals with an ID (Horner et al., 1990). Whether PBS is seen as consistent with ABA (Anderson & Freeman, 2000) or developing as a distinct discipline (Carr & Sidener, 2002; Kincaid, 2018), it has undoubtedly been, and arguably still is, substantially influenced by ABA (Johnston et al., 2006).

Carr and Horner (2007) drew our attention to the general movement from pathology-based models in favor of more contemporary positive models that emphasize environmental integrity and personal competence (Carr et al., 2002). Furthermore, Carr and Horner conceded that PBS benefits from many decades of scientific research in ABA, which has provided the conceptual framework for numerous assessment procedures, strategies, and interventions (Carr et al., 2002). Moreover, Filter et al. (2009) found that professionals who affiliated exclusively with either PBS or behavior analysis expressed common ground on some of the core elements of both. However, the same professionals expressed greater disagreement regarding the importance of service delivery methods, values, and research methods utilized. That said, Carr and Sidener (2002) seemed to conceptualize PBS as a value-driven behavior analytic service-delivery framework. Likewise, Wacker and Berg (2002) saw PBS as a service delivery model and outlined the value of PBS in promoting the palatability of ABA to the general population. It is interesting that a similar point is made by opponents of ABA (including some of those promoting the neurodiversity movement perspective) who imply a more cynical motive by depicting PBS as a Trojan horse, enabling ABA to be incorporated into schools under the more acceptable brand of PBS (Murray, 2020). However, in defense to such claims, PBS has also been proclaimed as being higher up the evolutionary scale than ABA within the context of ideological movements to promote inclusion/normalization, person-center values, and deinstitutionalization, originally in the field of ID (Knoster, 2018). In the UK, the PBS Competence Framework provides a guide to delivering best practice PBS:

PBS combines the technology of behavioral intervention with the values of normalization, human rights, and self-determination to deliver effective person-centered support for people whose behavior challenges. Crucially, these values inform both the way in which this technology is used and the outcomes that it is designed to achieve. (Positive Behavioral Support Coalition UK, 2015, p. 6)

If we accept the premise that ideological movements can be subject to arbitrary change and cross-cultural variances (Cascio et al., 2021; Grinker, 2020; Kirkham, 2017), then ideological values take precedence over science. As a result, interventions and research data can become filtered through this lens, potentially promoting unhelpful compromises in both intervention and research findings (Filter et al., 2009; Jackson & Panyan, 2002; Johnston et al., 2006).

In practice, PBS interventions follow the idea of what has become known as "wrap-around support," the proactive integration of services across multiple domains, including education, home, family, clinical and therapeutic interventions (Clark & Hieneman, 1999; Hieneman & Fefer, 2017; Raulston et al., 2019). However, Dillenburger et al. (2014) noted that although one intervention does not fit all, the authors also cautioned against an ill-defined multidisciplinary approach evolving into an eclectic "hodgepodge" of interventions consisting of many conceptual and procedural inconsistencies that potentially sideline the empirical evidence-based efficacy of ABA in favor of a “throw everything at it to see what sticks” approach. The question, therefore, is whether PBS, as defined by proponents (Carr et al., 2002; Horner et al., 2021), is methodologically and conceptually compatible with behavior analysis? Reid (2020) conceded that PBS and ABA are linked. However, he caveated this conclusion with the observation that there is no consistent agreement on the precise nature of that link, which he referred to as leaving lingering questions. For PBS practitioners, the potential for an eclectic mismatch of procedures that fall under the umbrella of PBS is significant, motivated at best by misguided propaganda or, at worse, commercially driven pseudo-science (Keenan, 2017; Leaf et al., 2022). As a result, PBS in the UK and Ireland often appears to have little empirical evidence of its engagement in the teaching of functional skills or utilizing skill sets inspired by ABA (Fennel & Dillenburger, 2018).

Working for PBS services within NI, two of the current authors have often received reports about professionals who experienced barriers to implementing skills-based interventions. To teach functional-based skills, the controlling environmental contingencies need to be managed. However, in our experience, purposively controlling or modifying these contingencies has been considered "manipulative" by those who subscribe to a different perspective under the guise of PBS. The result is that professionals have been forced to withdraw effective interventions with the result that levels of challenging behavior return to preintervention levels. In such examples, implementation of PBS has been diluted to only using antecedent interventions, whereas challenging behavior continues to be reinforced with no alternative behaviors taught. Matters are further compounded by the fact that PBS subscribes to the value of “self-determination,” which is inconsistent with the philosophical position of radical behaviorism (Berkholz et al., 2021; Rajaraman et al., 2023; Singer & Wang, 2009). Thus, the inconsistencies within and surrounding the PBS approach often mean that the “throw everything at it to see what sticks” approach that Dillenburger et al. (2014) warns against is often the reality of “PBS” within NI.

## Person-Centered Values

ABA, it could be argued, has always been in the truest sense a person-centered approach with its focus on measuring individual behavior across time and the use of single-case experimental designs to demonstrate control, as opposed to the aggregation of data across individuals (Cowan et al., 2017; Keenan & Dillenburger, 2011). In this way, behavior analysis takes into account individual variability (Johnston et al., 2019) and the ethos has always been to empower people, not normalize them, all the while retaining a perspective that is not ableist. As noted by Baer (2005):

The effectiveness of applied behavior analysis depends on *analysis.* When teaching techniques fail, which often happens with autistic children, that failure should be detected within a few days. . . . In ABA, a graph showing no progress in the last two weeks is always a powerful indicator of a need for analysis and changes in teaching technique reflecting that analysis. (p. 8)

Such analysis prevents unsubstantiated value judgements by the teacher, interventionist, or researchers alike. Moreover, rather than labelling the person or client as “aggressive,” for example, behavior analysts work to uncover the environmental contingencies which establish, shape, and maintain behavior (Fisher et al., 2021). Friman (2021) referred to this as the “circumstances view” of behavior, and neatly summarized the perspective by quoting a Catholic Priest, Father Flanaghan, who stated: "there is no such thing as a bad boy, only bad environment, bad modelling, and bad teaching" (Friman, 2021). The application of the circumstances view has significantly affected the lifestyle changes for people diagnosed with ASD, facilitating their inclusion within the community (Burtner, 2020). Is this not the epitome of the person-centered practice espoused by many other value-based approaches and organizations?

Friman (2021) noted that the normalization and deinstitutionalization movements have long availed of behavior technologies for those with mental health or developmental disabilities (see Ayllon & Michael, 1959; Wolf et al., 1963; Wolfensberger et al., 1972). However, PBS's overt promotion of these social/ethical concepts is a probable factor in its rapid dissemination and uptake by organizations within the ASD and ID care sector (Friman, 2021). Johnston et al. (2006) conceded that PBS has had a superior marketing strategy and that lessons could be learnt to promote the acceptance of ABA. However, Johnston et al. argued that the dissemination of PBS has not furthered the acceptance of ABA (Dillenburger et al., 2016; Johnston et al., 2006). On the contrary, the NAS contrast between PBS and ABA shows the damage being done to the uptake of ABA in the community when proponents of PBS denigrate ABA. Keenan (2017) likens the propagation of such misinformation to “fake news” with its focus on promoting PBS at the expense of the scientific methods used in ABA.

## Intervention Emphasis

Johnston et al. (2006) pointed to PBS’s overemphasis on antecedent elements and procedures with comparably less emphasis on the role of consequences. This is most evident with the increasing focus on broad ecological antecedent interventions such as inclusion and awareness of ASD with the aim of increasing societal acceptance and understanding, what Dunlap et al. (2008) refer to as “context” and “macro variables” (Dunlap et al., 2008, p. 693). Thus, the actual origins of challenging behavior could potentially be obscured by what Skinner (1953) refers to as the logical fallacy of *post hoc ergo propter hoc* (after this, therefore, because of this). For example, challenging behavior can often result in exclusion or restricted access to many community activities or facilities (Emerson & Bromley, 1995; McClean & Grey, 2007). It is conceivable that the PBS focus on these types of ecological or social value-based strategies could ultimately increase societal acceptance of a particular challenging behavior, thus facilitating greater levels of inclusion. However, in reality, the challenging behavior may continue to be emitted at the same level and severity if the maintaining variables have not been identified and manipulated to decrease its occurrence (Fisher et al., 2014). In other words, because a specific antecedent event takes priority, this does not mean that consequences should be relegated in importance in the PBS approach (Miltenberger, 2015). It is the correct balance within the contingent relations of the three-term contingency that determines the analysis of any procedure (Keenan & Dillenburger, 2018).

Many of the skill deficits observed in individuals with developmental disabilities and ID (e.g., language, social, and academic deficits) can also be found in many individuals diagnosed with ASD (see Matson et al., 1996). The acquisition or improvement of such skills would have a positive impact, not only for daily living skills but at the macro level allowing greater inclusion for autistic individuals (Friman, 2021). The beneficiary of the extensively researched and validated ABA technology is not just the person with ASD but also the parents or primary caregivers (Dogan, 2023; Sun, 2022). Healthy functional relationships are essential for all and, indeed, for wider society (Dogan et al., 2017). However, the elephant in the room is the question of whether or not a PBS practitioner can utilize such technology without full engagement with the principles and practices associated with behavior analysis? On the other hand, are such practitioners still practicing PBS if not applying skill development interventions? Without adequate training, the former would be unachievable and the latter indefensible.

## Training and Certification

Fischer et al. (2021) advocated evidence-based behavioral problem-solving for challenging behavior. They argued it would be remiss not to accompany this approach with the appropriate behavioral training. Although the Behavior Analyst Certification Board (BACB) and the United Kingdom Society for Behaviour (UK-SBA, n.d.) require standards of knowledge and skill-based technical requirements (BACB, n.d.; UK-SBA, 2023a, b), similar homogeneity seems relatively lacking in PBS. To date, PBS has no certification protocol, and there are no statutory training requirements to practice PBS (Cihon et al., 2020; McDaniel et al., 2017). Thus, anyone can claim to be a specialist and have knowledge of PBS, a reality confirmed by Horner and Kittelman (2021). This starkly contrasts with the academic and practical training criteria for ABA practitioners (Kazemi & Shapiro, 2013). Once behavior analytic certification is achieved, certificants are compelled to adhere to a code of ethics (Bailey & Burch, 2022), practice competency standards and obtain continuing education credits on a 2-year cycle (BACB, 2021), with a similar standard adopted by the UK-SBA (2023a, b).

It is paradoxical that proponents of PBS such as Horner et al. (1990) and Carr et al. (2002) acknowledged the need for all of its practices to be grounded in ABA, and they take the view that one cannot claim to be doing PBS without full engagement with the principles and practices associated with behavior analysis (Horner & Kittelman, 2021). This begs the question as to how a PBS practitioner in the UK or Ireland can effectively utilize behavior intervention procedures without appropriate scientific and technical training in behavior analysis (Tincani, 2007). The early innovators of PBS, such as Carr et al. and Horner et al., had the advantage of extensive training in the scientific foundations, philosophy, and systematic methodology of ABA with the associated certification by the BACB. Given Horner and Kittelman's (2021) stated importance of training in ABA, it behooves all PBS practitioners in the UK and Ireland to rectify the misrepresentation of ABA that appear on the NAS and BILD websites. In addition, there is a duty of care responsibility to correctly inform other organizations about the need for training in ABA in their guidelines of best practice on how to support ASD and ID individuals who engage in challenging behavior (Royal College of Psychiatrists, 2007). The current authors have come to the conclusion that this is all the more important now that in the UK and the Republic of Ireland, PBS is a mandatory framework to provide solutions for the challenging behavior of individuals with an ID or ASD diagnosis (Grey et al., 2016).

It is interesting that Skinner (1974) conceded that the precise terminology utilized by behavior analysis could be a potential variable in the lack of appeal of ABA to the general lay population. That is, the potential difficulty experienced in interpreting this scientific language may alienate the community to the benefits of ABA interventions or even produce animosity towards it (Bailey, 1991; Deitz & Arrington, 1983; Foxx, 1996; Lindsley, 1991; Marshall et al, 2023; O'Leary, 1984; Skiba & Deno, 1991). However, as with all sciences, a taxonomy is important. Without such a behavioral taxonomy, we risk unravelling the precision of its scientific language and replacing it with loose and subjective, often nondescript labels, thus compromising the validity and reliability of its applications and ultimately its efficacy (Keenan et al., 2010). This lack of understanding has been shown to extend beyond the lay population. Arntzen et al. (2010) found misconceptions in university students' and teachers' understanding of behavior analytic terminology, including significant confusion and conceptual misunderstandings (Lamal, 1995). Perhaps more concerning is that similar misconceptions have been found among professionals, most notably in direct care staff (Atun-Einy & Ben-Sasson, 2018; Donat & McKeegan, 1990). However, at the same time, as indicated by the ethics codes of both the BACB and the UK-SBA, behavior analysts are to use understandable language when communicating with clients, customers, and stakeholders. The misconceptions and misunderstandings of behavior analytic terminology among professionals highlights the difficulties one may face when attempting to implement scientific, analytic, and/or technical procedures that characterize the field of behavior analysis. This led Fennell and Dillenburger (2018) to argue for more emphasis on training professionals, such as teachers working with individuals diagnosed with ASD, in the fundamentals of behavior analysis.

In a review of training needed for the competent application of PBS, Mahon et al. (2022) acknowledged and highlighted the need for appropriate and effective staff training in challenging behavior and behavioral interventions. Mahon et al. called for more evidence-based studies of the efficacy of staff-based PBS instruction. They reiterated a call by Bradly and Nathan (2019) for more experimentally rigorous designs to identify the efficacy of PBS training (Bradly & Nathan, 2019; Mahon et al., 2022). However, if PBS practitioners seek behavior analytic advice from a credentialed professional behavior analyst or even work to implement the advice, at what stage could they be said to be practicing ABA? At what stage does an intervention design stop being referred to as PBS or an eclectic approach?

## Aversive Procedures

PBS practitioners reject aversive and restrictive practices (Horner et al., 1990; Knoster, 2018; Positive Behavioral Support Coalition UK, 2015). As stated, part of the development of PBS was the reaction to the historically perceived overuse of aversive procedures and ABA’s focus on consequent-based interventions directed at individuals diagnosed with ID, with little regard for the individual's dignity (Horner et al., 1990). Although this perception was compounded by a number of prominent abuse scandals in the late 1980s and early 1990s, most notably the Sunland Miami residential unit in the State of Florida, these incidents were more about the poorly informed application of behavior modification (Bailey & Burch, 2022). Bailey and Burch (2022) linked such abuses to an expectation of quick, easy fixes by poorly trained staff fueled by previous legitimate successes (e.g., Ayllon & Michael, 1959; Burchard & Tyler, 1965; Lovaas et al., 1965; Wolf et al., 1967). The contention that behavior analysts overused or misused aversive procedures and punishment is a post hoc value judgement that is contested (Johnston et al., 2006; Leaf et al., 2022). It could be contended that PBS makes the same erroneous assumptions made by some social movements for autism rights by disproportionately focusing on historical practices and reviewing them under contemporary social standards and knowledge (Furman & Lepper, 2018; Dillenburger & Keenan, 2023; Leaf et al., 2022). Furthermore, the focus on removing coercion or punitive practices has historical precedent in the uptake of behavior analysis well before PBS emerged (Bailey & Burch, 2022). In effect, the call from PBS advocates is a reminder not to forget their roots in ABA as outlined by its founders.

A frequently cited example of the use of aversives is the clinical and research practices of Ivor Lovaas in the Young Autism Project (YAP), which spanned over 20 years (Lovaas et al., 1973). Aversive procedures demonstrated quantifiable reductions of intensity and frequency in individuals emitting life-endangering, self-injurious, or severe obsessive, repetitive behaviors. It is controversial that electric shock was utilized initially (Smith & Eikeseth, 2011), with Lovaas later substituting shock for a spank (Lovaas, 1987). However, YAP's own guidelines aimed for a ratio of reinforcement to punishment at the very most to be 100:1 (Leaf & McEachin, 2016). Lovaas was undoubtedly a pioneer in the field who brought meaningful change to the lives of many children diagnosed with autism (Leaf et al., 2022). Before his seminal work, there was a general perception that autistic individuals were "beyond help" (i.e., incapable of change; Eikeseth, 2001). The fact that aversive procedures were used relatively more frequently in the past says more about the moral climate at that time. The Equal Protection from Assault (Scotland) Act 2019 (see Rogers & Thomas, 2022), covering the principle of reasoned chastisement, outlawed all physical punishment, including smacking, for children under 16 as of November 7, 2020. This removes the caveat of "reasonable punishment." Although Scotland was the first part of the UK to legislate for this, the Welsh Assembly followed suit on March 21, 2022 (Rogers & Thomas, 2022) Likewise, in England and NI, legislation is currently under review with a view to making similar legislative changes (Rogers & Thomas, 2022). However, physical punishment by professionals such as teachers, nursery workers, care workers, or babysitters has been illegal since 1986 in the UK and from 2003 in NI (Speyer et al., 2021). Members of the Association for Behavior Analysis International (ABAI) recently voted in their opposition to the use of the punishment procedure of using contingent electric skin shock under any condition, no matter how extreme the behavior (Perone et al., 2022).

Mainstream ABA has long since embraced socially valid aspects of its interventions (Metz et al., 2005). Indeed, one of the founding fathers of ABA, Don Baer, said the following: "Everything I have described as typical ABA programming does not evoke negative emotions” (Baer, 2005, p. 14). This viewpoint has been enshrined in the BACB's *Ethics Code for Behavior Analysts*, which emphasizes "providing effective treatment" (code 2.01, p. 10) and "minimizing risk of behavior change" (code 2.15, p. 12); the BACB would only recommend "restrictive or punishment based procedures only after demonstrating that desired results have not been obtained using less intrusive means, or when it is determined by an existing intervention team that the risk of harm to the client outweighs the risk associated with the behavior change" (code 2.15, p. 12). Likewise, the UK-SBA states that "All UK-SBA registrants have signed up to our ethical code, and any registrant found to be using harmful, degrading, painful, or dehumanizing punishment procedures will be removed from our register" (UK-SB 2023a, b, p. 1). That said, punishment is a well-defined principle within ABA. The behavior analytic definition of punishment relates to behavior change i.e., a consequence of behavior that reduces the future probability of that behavior (Azrin, 1970). This can often differ considerably from the layperson’s perception of what constitutes punishment, which is often conflated with coercive and punitive techniques within context of social or personal retribution.

However, using the behavioral definition, even a kiss could be categorized as a punisher for an individual if it decreases the target behavior (Foxx, 1985). If an ABA practitioner discusses punishment contingencies with a fellow behavioral scientist, the common language facilitates objective comparisons and eliminates misunderstandings. It should also be noted that punishment contingencies operate independently of the science of behavior analysis and thus have been operational long before the conception of ABA (Skinner, 1990). Consider a person who may have first tried to touch an open flame and experienced the aversive consequences (i.e., pain) which reduced the likelihood of them engaging this behavior in the future. The only thing that is new is a better understanding how to categorize the effects of this interaction using the science of behavior analysis (Skinner, 1990).

It is the science of behavior analysis which first discovered and demonstrated the functional and possible adverse side effects of punishment; inducing escape and avoidance (Mayer et al., 1968), aggression (Ulrich et al., 1969), stress and anxiety (Sidman, 1989), and negatively reinforcing the behavior of the punishing agent (Lerman & Vorndran, 2002). However, according to recent empirical research, the seemly ubiquitous adverse side effects of punishment may not be as widespread as previously believed (Fontes & Shahan, 2021).

Dillenburger and Keenan (1995) argued that the legal prohibition of such aversive punishment procedures is not enough, and that ABA has at its disposal a range of scientifically validated alternative techniques to tackle CB that do not include aversive procedures (LaVigna et al., 2022). However, if, as we noted, PBS practitioners are prevented from implementing consequence-based interventions, aimed at teaching functional alternative behaviors, there are serious implications for clients and various stakeholders.

## Science and PBS

It is the current authors concern that with the importance allocated by PBS to broad issues such as inclusion and normalization, it becomes difficult to separate it from social or political movements, risking the uncritical adaption of subjective philosophical assumptions at the expense of objective science. As Cooper et al. (2020) reminded us, science is not the tool for validating treasured beliefs or our preferred versions of how things should be. The inherent ethical danger of such prioritization is that practitioners may ultimately be imposing their own cherished values or beliefs on the individuals they are there to assist (Baer, 1998). There has been a push by some exponents within PBS to adopt more flexibility in its application of scientific procedures (Scott et al., 2009). The rationale offered details of practical problems such as the resources (e.g., time) required to conduct assessments (e.g., functional analysis) and the difficulties in controlling variables within the community setting (Desrochers et al., 1997; Kern et al., 2006). However, Hanley (2012), for example, rejected such difficulties as myths and demonstrated the practical utility of scientific procedures such as experimental functional analysis in applied settings (Jessel et al., 2022). Although, it should be noted that the validity of such experimental functional analyses is not universally accepted within the behavior analytic community (Fisher et al., 2016; Tiger & Effertz, 2021).

Regardless, the implication is that PBS is “ABA light,” in other words, containing less content, being relatively less complex, or less technical (Ferguson et al., 2019). It is unclear whether PBS can be defined as an application, an applied science, a technology, a collection of procedures, or simply a framework for delivery. Which of these definitions’ proponents deem most relevant can vary and some even incorporate all these components (Kincaid, 2018). Although perhaps no single definition of PBS would be universally acceptable to all PBS or ABA practitioners, it is often referred to as being a multicomponent framework (Gore et al., 2013). However, in the current reviewed literature on PBS the parallels with components of ABA seem unavoidable. Any analysis of the identity of PBS ultimately reverts to conceptual, procedural, and ethical comparisons with ABA past and present.

## Conclusion

The current analysis is based on writings from prominent proponents of PBS (e.g., Allen et al., 2005; Carr et al., 2002; Horner et al., 1990; Horner et al., 2021) and our own “lived experience in working in a PBS environment in NI.” Many other publications on ABA and PBS revealed that what variance there is on the surface, at times, appears small and no more than the debates within the disciplines that make up ABA (Weiss et al., 2010). In fact, when you look at Skinner’s criticism on the use of aversives (Griffin et al., 1988), it can easily be argued that PBS has simply voiced its opposition to those who have strayed too far from his vision. Harvey (1973) expressed Skinner’s vision like this:

We cannot imagine how a nonpunishing world would work, even though it is clear that the punishing world we have does not work. That is, *most* of us cannot imagine what a nonpunishing world would be like. A minority among us can, however. Its members are adherents of a new behaviorism with a new non-Benthamite psychology, one that claims to rest upon solid scientific foundations rather than merely superficial common-sense postulates. This is the psychology of operant conditioning, invented (discovered) by B. F. Skinner. (p. 4)

Is there enough to constitute the separation of PBS from ABA as a distinct science? The Carr et al. (2002) brand of PBS has close conceptual and procedural links that appear to display only moderate differences from ABA. However, Carr et al. viewed PBS differently enough to consider it a new science. There is less consensus on how close this relationship should be in the future. The appeal of a relatively newly marketed and rapidly growing approach which distances itself from a more established approach may add to the allure of PBS (Johnston et al., 2006). The PBS proponents are not immune to the environmental consequences of conditioned reinforcers such as academic acclaim and financial and social reinforcement (Keenan et al., 2010). It is interesting that Skinner (1988) likened the appropriation of the principles of behavior analysis to the actions of cuckoos—birds that lay their eggs in other birds' nests, so the host bird does all the work in rearing its offspring. Is PBS the cuckoo in the nest? Such depiction implies that the ideological purity of behavior analysis is threatened by such diversification of the field although these same proponents are still able to reap the benefits of the knowledge gained in the field. Friman (2021) suggested the possible existence of an evolutionary instinct that leads an organism to interpret differences as potentially threatening hence the universal tendency to attack it. In doing, Friman related this as a possible factor in the forceful criticisms by some "conventional behavior analysis" of PBS. This rubric assumes that there is considerable similarity between PBS and ABA, with only modest differences between the fields and that the attack is not directed at ABA. Others are more concerned about the potential increasing divisive effect that the debate is having (Weiss et al., 2010). In reviewing the literature on the difference between PBS and ABA, we believe there is a temptation to overstate the case in either direction. At the same time, we might argue that there is an element of political naivety on the part of PBS proponents. This is evident outside of the United States in countries where training opportunities in ABA have yet to be established (Keenan et al., 2023; Kingsdorf & Pancocha, 2023; Sivaraman & Fahmie, 2020).

How can policymakers decide between investing in ABA or PBS training in such a country? This becomes a serious issue when proponents of PBS denigrate ABA, as happens in the UK (BILD, 2022). In effect, this defamation of ABA misleads policymakers into concluding that the breakthrough at the state level in the independent support for ABA is to be considered irrelevant for policy decisions in relation to autism service provision. Without an existing infrastructure in a country to provide training in ABA, the consequence is that this makes it more difficult for professionals to develop the science in their own countries. Those affected most are the parents, caregivers, and indeed the ASD individuals (Dillenburger et al., 2010). Are these individuals who are in need of assistance just to be collateral damage? Are both service providers and service users being deprived of effective interventions and knowledge born out of the science of ABA? It is the opinion of the current authors that when PBS emphasizes a value-driven position, this implies an air of ethical and moral superiority. However, general ethical principles such as the pursuit of excellence, accountability, and do no harm (Keith-Spiegel & Koocher, 1998) which are included in and inspire many ethical codes, including behavior analytic and medical codes, seem inconceivable without the prerequisite knowledge and training before any intervention. Bailey and Burch (2022) identified insufficient knowledge and training as a significant factor in the drift from treatment to abuse in scandals such as the Sunland residential home (McAllister, 1972). The consequence of such a scandal has changed ABA practice, leading to statutory requirements for certification, supervision, and training before practice (Bailey & Burch, 2022). Such consistency in a flooded marketplace of interventions and treatments for autism can only inject more clarity in a melee of proposed experts and specialists in the field.

Keenan (2017) suggested a possible solution regarding productive comparisons and the current confusion of clients who need to invest in effective strategies and treatment packages. Historical reviews can easily connect PBS to ABA (Carr & Sidener, 2002; Gore et al., 2013), as they can with Early Intensive Behavioral Intervention (Dunlap & Fox, 2009; Eldevik et al., 2009), Picture Exchange Communication System (Bondy & Frost, 1994), and Pivotal Response Training (Koegel et al., 1996). That being so, it makes perfect sense to reflect these historical connections by anchoring their acronyms with ABA itself as in ABA: PBS, ABA: EIBI, ABA: PECS, ABA: PRT, respectively. Without such a nomenclature, it would not be inconceivable for the short-sightedness of PBS advocates and others who denigrate ABA, along with the misunderstandings promulgated by neurodiversity arguments, to coalesce into a movement that prevents the development of ABA in other countries. Although some of the arguments made in this paper have been made before, they seem to have been unsuccessful in arresting the continued fragmentation of a science. Our goal in this article has been to rekindle discussion because spreading misinformation about ABA seems to be fair game for those who experience no consequences for doing so.
