# Correction: A Discussion of Positive Behavior Support and Applied Behavior Analysis in the Context of Autism Spectrum Disorder in the UK and Ireland

**DOI:** 10.1007/s40617-024-01029-6

**Published:** 2024-12-10

**Authors:** David Stalford, Scott Graham, Michael Keenan

**Affiliations:** Ulster University, Coleraine, Londonderry, Northern Ireland

**Correction: Behavior Analysis in Practice (2024) 17:442–455**

10.1007/s40617-023-00905-x

The original article has been corrected. The incorrect reference “Larsson, E. V. (2013). Is applied behavior analysis (ABA) and early intensive behavioral intervention (EIBI) an effective treatment for autism? A cumulative review of impartial reports. Autism, 27(1), 168–179. Retrieved December 20, 2022, from https://www.semanticscholar.org/paper/Is-Applied-Behavior-Analysis-(ABA)-and-Early-(EIBI)-Larsson/80f58225e081e55b83e0a5472f833196f71d4bfc” has been changed to “Larsson, E. V. (2021). Are Applied Behavior Analysis (ABA) and Early Intensive Behavioral Intervention (EIBI) Effective, Medically Necessary Treatments for Autism? A Cumulative History of Impartial Independent Reviews. Cambridge Center for Behavioral Studies. Web publication available at: https://behavior.org/wp-content/uploads/2017/06/Larsson2021AreABAandEIBIEffectiveTreatmentsforAutismReviews.pdf”.

